# Individual participant data meta‐analysis of continuous outcomes: A comparison of approaches for specifying and estimating one‐stage models

**DOI:** 10.1002/sim.7930

**Published:** 2018-08-13

**Authors:** Amardeep Legha, Richard D. Riley, Joie Ensor, Kym I.E. Snell, Tim P. Morris, Danielle L. Burke

**Affiliations:** ^1^ Centre for Prognosis Research, Research Institute for Primary Care & Health Sciences Keele University Keele UK; ^2^ London Hub for Trials Methodology Research MRC Clinical Trials Unit at UCL London UK

**Keywords:** continuous outcomes, estimation, individual participant data, IPD, meta‐analysis

## Abstract

One‐stage individual participant data meta‐analysis models should account for within‐trial clustering, but it is currently debated how to do this. For continuous outcomes modeled using a linear regression framework, two competing approaches are a stratified intercept or a random intercept. The stratified approach involves estimating a separate intercept term for each trial, whereas the random intercept approach assumes that trial intercepts are drawn from a normal distribution. Here, through an extensive simulation study for continuous outcomes, we evaluate the impact of using the stratified and random intercept approaches on statistical properties of the summary treatment effect estimate. Further aims are to compare (i) competing estimation options for the one‐stage models, including maximum likelihood and restricted maximum likelihood, and (ii) competing options for deriving confidence intervals (CI) for the summary treatment effect, including the standard normal‐based 95% CI, and more conservative approaches of Kenward‐Roger and Satterthwaite, which inflate CIs to account for uncertainty in variance estimates. The findings reveal that, for an individual participant data meta‐analysis of randomized trials with a 1:1 treatment:control allocation ratio and heterogeneity in the treatment effect, (i) bias and coverage of the summary treatment effect estimate are very similar when using stratified or random intercept models with restricted maximum likelihood, and thus either approach could be taken in practice, (ii) CIs are generally best derived using either a Kenward‐Roger or Satterthwaite correction, although occasionally overly conservative, and (iii) if maximum likelihood is required, a random intercept performs better than a stratified intercept model. An illustrative example is provided.

## 1 INTRODUCTION

Individual participant data (IPD) meta‐analysis involves obtaining and then synthesizing raw individual‐level data from multiple related studies, to produce summary results that inform clinical decision making.^1^ The IPD approach is increasingly popular and has many potential advantages over a traditional meta‐analysis of published aggregate data, such as increased power to detect treatment‐covariate interactions and avoiding reliance on published results.^2^

Statistical methods to perform an IPD meta‐analysis involve either a one‐stage or two‐stage approach.^3^ Generally, these approaches give very similar meta‐analysis results, especially when they use the same modeling assumptions and/or estimation methods.(4, 5) However, the one‐stage approach has become increasingly popular over the past decade.^6^ It conveniently allows all studies to be analyzed simultaneously and avoids the assumption of normally distributed study effect estimates with known variances that is usually made in the second stage of the two‐stage approach. It also allows greater flexibility of parameter specification over the two‐stage approach.^6^

When conducting a one‐stage IPD meta‐analysis, it is important to account for clustering of participants within studies, to correctly condition an individual's response to the study they are in. Ignoring clustering and analyzing IPD as if coming from a single study can result in misleading conclusions. For example, Abo‐Zaid et al^7^ showed that family history of thrombophilia was statistically significant as a diagnostic marker of deep vein thrombosis when clustering was accounted for (odds ratio = 1.30; 95% confidence interval (CI): 1.00, 1.70; p value = 0.05) but not when clustering was ignored (odds ratio = 1.06; 95% CI: 0.83, 1.37; p value = 0.64). While it is well established that clustering should be accounted for, it is debatable exactly how this should be done. In particular, there are two competing approaches to account for clustering in a one‐stage model: a stratified intercept or a random intercept. The stratified approach involves a separate intercept term being estimated for each study; thus, if there are 10 studies, 10 intercept terms would be estimated (one for each study). In the random intercept approach, the intercepts are assumed to be drawn from some distribution, typically normal with an underlying mean value and variance. The advantage of the stratified intercept approach is that it makes no assumptions about the distribution of intercepts across studies. In contrast, the advantage of the random intercept approach is that it requires fewer parameters to be estimated.

In this article, we evaluate through an extensive simulation study the impact of using either the stratified or random intercept approach on the statistical properties of the summary treatment effect estimate (for example, in terms of bias, precision, mean square error (MSE), and coverage). This is considered in the context of randomized trials with a continuous outcome and a 1:1 treatment:control allocation ratio, assuming either common or random treatment effects across trials. Two further aims are to (i) compare competing estimation options for the one‐stage models, including maximum likelihood (ML) and restricted maximum likelihood (REML) and (ii) compare competing options for deriving confidence intervals for the summary treatment effect, including the standard normal‐based 95% CI, and (for REML, but not ML estimation) the Kenward‐Roger (KR)^8^ and Satterthwaite^9^ corrections that inflate confidence intervals to account for uncertainty in variance estimates.

This paper is structured as follows. In Section 2, we introduce the two competing one‐stage IPD meta‐analysis models of interest that account for clustering, as well as the competing estimation and CI derivation options. In Section 3, we outline how the simulation study was conducted and present the results, and in Section 4, we provide a real example to illustrate the methods considered. Finally, in Section 5, we conclude with a discussion of the key findings and limitations and offer a recommendation for those conducting one‐stage IPD meta‐analysis of randomized trials with 1:1 treatment:control allocation ratio and with a continuous outcome.

## 2 INTRODUCING DIFFERENT MODEL SPECIFICATION AND ESTIMATION OPTIONS

Consider that IPD have been obtained from *i* = 1 to *K* related randomized trials, each investigating a treatment effect based on a continuous outcome *Y* (say, blood pressure); that is, the mean difference in outcome value between a treatment and a control group. Suppose that there are *n*_*i*_ participants in trial *i*. Let *Y*_*Fi j*_ be the end‐of‐trial (F used to denote final) continuous outcome value, for participant *j* in trial *i*, and *Y*_*Bi j*_ (B to denote baseline) be the pre‐treatment outcome value. Let *treat*_*i j*_ take the value 1 or 0 for participants in the treatment or control group, respectively.

Given such IPD, there are several ways in which researchers can use a one‐stage meta‐analysis to model the summary treatment effect across trials. We focus initially on presenting one‐stage analysis of covariance (ANCOVA) mixed models, which either use a stratified intercept or a random intercept to account for clustering of participants within trials. We also assume a random treatment effect since heterogeneity is usually expected.

### 2.1 Model (1): stratified intercept

With the following approach, a stratified intercept is used to account for within‐trial clustering.

(1) $$\begin{array}{cc} Y_{\mathrm{Fij}} & =\beta_{i}+\lambda_{i}\left(Y_{\mathrm{Bij}}-{\bar{Y}}_{\mathrm{Bi}}\right)+\left(\theta +u_{i}\right){\text{treat}}_{\mathrm{ij}}+e_{\mathrm{ij}}\\ & \;u_{i}∼N\left(0,\tau^{2}\right)\\ & \;e_{\mathrm{ij}}∼N\left(0,\sigma_{i}^{2}\right) \end{array}$$

Here, *β*_*i*_ denotes the intercept term for trial *i* (expected final outcome value for participants in the control group in trial *i* who have the mean baseline outcome value), and the distinct intercept for each trial is used to account for within trial clustering. The term *λ*_*i*_ denotes a trial‐specific adjustment term for the baseline outcome value (here, centered at the mean for each trial ( ${\bar{Y}}_{\mathrm{Bi}}$) to aid interpretation of the trial‐specific intercepts). For example, when there are *K* = 10 trials, there would be 10 *β*_*i*_ terms and 10 *λ*_*i*_ terms. Of main interest is an estimate of the model parameter θ, as this denotes the summary (average) treatment effect. The random effect, *u*_*i*_, indicates that the true treatment effects in each trial are assumed to arise from a distribution of true effects with mean *θ* and between‐trial variance *τ* ^*2*^. This assumption could be constrained if considered appropriate, with a common (fixed) treatment effect (ie, constrain *τ* ^*2*^ = 0). Lastly, $\sigma_{i}^{2}$ denotes a distinct residual variance per trial.

The flexibility of the one‐stage IPD approach allows us to make further modifications by considering, for example, a common baseline adjustment term (ie, *λ*_*i*_= *λ*) across trials, or common residual variances (ie, $\sigma_{i}^{2}=$ *σ* ^*2*^) if necessary(5, 10, 11); however, this should be justified (eg, based on computational reasons or estimation problems), and sensitivity analysis to the choice of assumptions is often sensible.

### 2.2 Model (2): random intercept

When there are a large number of trials to be synthesized, a stratified intercept approach to clustering can be computationally intensive (as Equation (1) requires estimation of 3* K + *2 parameters).^4^ An alternative approach for dealing with clustering, which is preferred by some researchers,^12^ is to use a random intercept term.

(2) $$\begin{array}{cc} Y_{\mathrm{Fij}} & =\left(\beta +u_{1i}\right)+\lambda_{i}\left(Y_{\mathrm{Bij}}-{\bar{Y}}_{\mathrm{Bi}}\right)+\left(\theta +u_{2i}\right){\text{treat}}_{\mathrm{ij}}+e_{\mathrm{ij}}\\ & \;u_{1i}∼N\left(0,\tau_{\beta }^{2}\right)\\ & \;u_{2i}∼N\left(0,\tau^{2}\right)\\ & \;e_{\mathrm{ij}}∼N\left(0,\sigma_{i}^{2}\right) \end{array}$$

Parameters are as in Equation (1), except that within‐trial clustering has now been accounted for by a random (instead of stratified) intercept term, with $\;\tau_{\beta }^{2}\;$ denoting the between trial variance in the intercept about the mean intercept (*β*). Equation (2) assumes independence of the two random effects (ie, a covariance of zero), but their correlation could be accounted for assuming a bivariate random effect distribution; indeed, this might be of special interest when evaluating the relationship across trials of mean baseline in the control group and true treatment effect.^13^

Compared to Equation (1), the number of parameters to be estimated has been reduced, with only *β* and *τ*_*β*_ for the intercept, instead of *K* separate terms. Therefore, fewer estimation problems might be anticipated than in Equation (1). On the downside, Equation (2) makes a strong and potentially unnecessary assumption that control group means are drawn from a normal distribution with a common mean and variance. Furthermore, the estimation of an additional random effect term might increase computational intensity.

### 2.3 Options for estimation and CI derivation

The parameters in models (1) and (2) are typically estimated using either a ML or REML approach. ML is known to produce downwardly biased estimates of between trial variance when there are few trials,(14, 15, 16) whereas REML addresses the downward bias and is thus generally preferred.(17, 18)

In addition to competing options for model parameter estimation, there are also competing options to subsequently derive (1 − *α*)100% CIs for the true summary treatment effect (*θ*). Standard CIs are based on large‐sample inference and assume $\hat{\theta }$ is approximately normally distributed:

(3) $$\hat{\theta }\pm z_{1-\frac{\alpha }{2}}\sqrt{\mathrm{Var}\left(\hat{\theta }\right)},$$

where $\hat{\theta }$ is the estimate of *θ*, $\mathrm{Var}\left(\hat{\theta }\right)$ is its variance, and $z_{1-\frac{\alpha }{2}}$ is the upper $1-\frac{\alpha }{2}$ quantile of the standard normal distribution. This standard approach may produce CIs that are too narrow, as $\mathrm{Var}\left(\hat{\theta }\right)$ does not account for the uncertainty in the estimate of the between trial variation of $\hat{\theta }$.(4, 18)

To address this, more conservative options are available based on small‐sample inference, which define the uncertainty around $\hat{\theta }$ using approximations based on a *t*‐distribution, such as the KR^8^ and Satterthwaite^9^ corrections, which are also known as denominator‐degrees‐of‐freedom adjustments.

The KR corrected (1 − *α*)100% CI is given by

(4) $$\hat{\theta }\pm t_{\upsilon ;1-\frac{\alpha }{2}}\sqrt{{\mathrm{Var}}_{KR}\left(\hat{\theta }\right)},$$

where $\hat{\theta }$ is as before, but now a bias‐adjusted (inflated) variance $\left({\mathrm{Var}}_{KR}\left(\hat{\theta }\right)\right)$ is used, and $t_{\upsilon ;1-\frac{\alpha }{2}}$ (the upper 1− $\frac{\alpha }{2}$ quantile of the *t*‐distribution with an adjusted degrees of freedom, υ) instead of $z_{1-\frac{\alpha }{2}}$.

For a single parameter of interest (as in our case), the Satterthwaite corrected (1 − *α*)100% CI is given by

(5) $$\hat{\theta }\pm t_{\upsilon ;1-\frac{\alpha }{2}}\sqrt{\mathrm{Var}\left(\hat{\theta }\right)},$$

where $t_{\upsilon ;1-\frac{\alpha }{2}}$ is as in the KR correction, but the original (unadjusted) variance of $\hat{\theta }$ is used. Note that, while the denominator degrees of freedom calculated from the KR and Satterthwaite corrections are the same for single hypothesis tests, the KR correction uses a bias‐adjusted variance; therefore, CIs derived using Equations (4) and (5) will potentially differ, with the one using the KR correction (Equation (4)) leading to slightly wider intervals.^19^

Although Schaalje et al^20^ recommend KR over Satterthwaite in special cases when the sampling distribution of the test statistic is known, there remains debate over the best method, and a lack of literature in this area in regard to IPD meta‐analysis for estimation of a parameter of interest.

## 3 SIMULATION STUDY

We now perform a simulation study to examine the statistical performance of the summary treatment effect estimate ( $\hat{\theta }$) from a one‐stage IPD meta‐analysis across a range of scenarios. Our aim is to assess the different model specifications, parameter estimation methods and CI derivation options described in Section 2. That is, we compare the following: stratified or random intercept specifications; ML or REML estimation options; and, for REML estimation, 95% CIs based on asymptotic formula (Equation (3)) or with either KR or Satterthwaite corrections (Equations (4) and (5), respectively)).

### 3.1 Methods

Provided is a step‐by‐step guide to our simulation study. For simplicity, and to considerably speed up the many simulations, we removed the baseline adjustment term in models (1) and (2), such that it does not exist in any of the data generating mechanisms or models fitted in our simulations. In other words, we generate data without baseline imbalances and thus analyze the data according to a final score IPD meta‐analysis model, which is appropriate in this situation_._ 21 For similar reasons of simplicity and computational complexity, we assumed a common residual variance across trials (both in data generation and models fitted). Extension to different residual variances is considered in our discussion (Section 5). To inform the true parameter values for the simulation, we used a previous IPD meta‐analysis of treatment for lower blood pressure outcomes.^22^

All analyses were conducted using Stata v.14.2 (Stata Corporation, TX, USA).^23^

#### 3.1.1 Scenario 1 (base case)

The simulation process is now explained, in the context of an initial base case scenario with IPD from 10 trials and a relatively simple data generating mechanism. Extensions to other more complex scenarios are described afterwards.

*Step 1: Data generating mechanism for one IPD meta‐analysis of 10 trials*

Consider that an IPD meta‐analysis of *i* = 1 to *K* related trials is of interest, with the goal to summarize a treatment effect on a continuous outcome. To generate such data for the base case of this simulation study, we started by setting the number of trials, *K*, to 10. We set a fixed number of participants, *n* = 100 in each trial, and assumed a fixed randomization of 1:1 in each trial; that is, on average, 50% of participants within any given trial are allocated to a treatment group, and the remaining 50% to a control group. This gave us a *trial*_*i*_ (trial 1/0 indicator) and *treat*_*ij*_ (treatment group 1/0 indicator) value for each of 100 participants in each of 10 trials.

Next, based on the previous meta‐analysis,^22^ we set the true parameter values for this simulation to be as follows: θ = −9.66 (summary treatment effect; negative value favors treatment group), *τ*^2^ = 7.79 (between trial variation in the treatment effect), *β* = 159.73 (mean blood pressure response in control group), $\tau_{\beta }^{2}$ = 233.99 (between trial variation in the intercept), and *σ* ^*2*^ = 333.74 (residual variance).

We then used these parameter values to generate further terms, beginning with using *σ* ^2^ to generate an error term *e*_*i j*_, for the *j*th participant from the *i*th trial

(6) $$e_{\mathrm{ij}}∼N\left(0,\sigma^{2}\right).$$

Then, we generated the trial level values for the random parts of the intercept and treatment effect terms, *u*_1*i*_ and *u*_2*i*_, respectively,

(7) $$\begin{array}{c} u_{1i}∼N\left(0,\tau_{\beta }^{2}\right)\\ u_{2i}∼N\left(0,\tau^{2}\right).\; \end{array}$$

Finally, with all the parameters defined (*β*, *u*_1*i*_, θ, *u*_2*i*_, *treat*_*i j*_, and *e*_*i j*_), we generated the end‐of‐trial continuous outcome value *Y*_*Fi j*_, under the random intercept model (2) (with no baseline adjustment term and assuming a common residual variance)

(8) $$Y_{\mathrm{Fij}}=\left(\beta +u_{1i}\right)+\left(\theta +u_{2i}\right){\text{treat}}_{\mathrm{ij}}+e_{\mathrm{ij}}.$$

This gave one complete IPD meta‐analysis dataset of 1000 total participants, containing 100 participants in each of 10 trials, consisting of the following data for each individual: a trial indicator (*trial*_*i*_), a treatment group indicator (*treat*_*i j*_), and an end‐of‐trial continuous outcome value (*Y*_*Fi j*_).

*Step 2: Model fit and replication*

**Figure 1:**
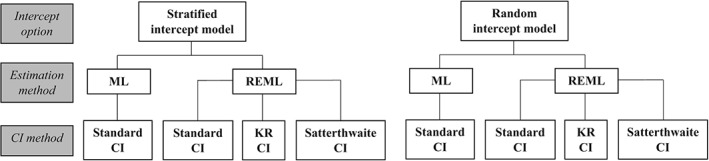
Flow diagram of possible combinations of intercept option, estimation, and CI methods. CI, confidence interval; KR, Kenward‐Roger correction; ML, maximum likelihood estimation; REML, restricted maximum likelihood

Using the generated data, we fitted a stratified intercept model (1) and a random intercept model (2) (without the baseline adjustment term and assuming a common residual variance) separately to this simulated IPD, under all the combinations of estimation and CI derivation methods outlined in Section 2. Figure 1 provides a flow diagram summarizing the possible combinations. Each time a model was fitted (under a particular combination of estimation and CI derivation methods), we stored the following: the summary treatment effect estimate, $\hat{\theta }$; its corresponding 95% CI; a binary indicator variable for coverage of $\hat{\theta }$ (ie, the value 1 if the 95% CI of $\hat{\theta }$ contained the true θ, and 0 otherwise); estimates of any variance parameters; model run time (from start of model fit to end of post estimation); and model convergence (1/0 for convergence within 100 iterations/nonconvergence, respectively).

For each model (stratified or random intercept) fitted to the data, this enabled us to obtain two estimates of θ (one each for the models fitted using ML and REML estimation, respectively) and four 95% CIs for $\hat{\theta }$ (one for ML estimation with a standard CI derivation, and then one each for REML estimation with the standard, KR‐corrected, and Satterthwaite‐corrected CI derivations).

Step 3: Simulation replications

Steps 1 and 2 were repeated until 1000 IPD meta‐analysis datasets had been generated using the true parameter values and procedure as outlined thus far, followed by application of the various intercept option, estimation, and CI methods to each of the 1000 replicated datasets (note: 1000 simulations were chosen to give a Monte Carlo error of 0.7% on a coverage of 95%).

Step 4: Summarizing performance

Using the results obtained after Step 3, the statistical properties of $\hat{\theta }$ under the different model specification and estimation options were assessed by summarizing the 1000 results obtained using the following metrics: mean percentage (%) bias, empirical standard error (SE), MSE, coverage (separately for each CI method), convergence, and mean run time (separately for each CI method). Additionally, we considered the median percentage bias in the heterogeneity (τ ^2^) of the true treatment effects also. Definitions of these performance measures are provided in Web Appendix A.

#### 3.1.2 Extended set of 38 scenarios changing number of trials, participants, between‐trial distributions, and data generating mechanisms

The base case scenario defined in Section 3.1.1 was extended to further settings, leading to an extensive range of 38 scenarios in total (see Table 1), which we now summarize.

**Table 1:** Summary of the different simulation scenarios*

Scenario	Data Generation Details	Modification From Base Case Scenario
**Base Case**	(i) Number of trials, K = 10	‐
	(ii) Number of participants in trial i, n_i_ = 100 (fixed across all trials)	
	(iii) Fixed treatment exposure of 50%	
	(iv) θ = −9.66 (summary treatment effect; negative value favors treatment group)	
	(v) τ ^2^ = 7.79 (between trial variation in θ)	
	(vi) β = 159.73 (mean response in control group)	
	(vii) τ_**β**_^2^ = 233.99 (between trial variation in β)	
	(viii) σ ^2^ = 333.74 (residual variance)	
**A1**	Same as base case, except changed (i)	K = 5
**A2**	Same as base case, except changed (i)	K = 20
**B1**	Same as base case, except changed (ii)	n_i_ ∼U(30, 1000)
**B2**	Same as base case, except changed (ii)	n_i_ ∼U(30, 100) for trials 1 to 5,
		n_i_ ∼U(900, 1000) for trials 6 to 10
**B1‐A1**	Same as base case, except changed (i) and (ii)	K = 5 and n_i_ ∼U(30, 1000)
**B1‐A2**	Same as base case, except changed (i) and (ii)	K = 20 and n_i_ ∼U(30, 1000)
**B2‐A1**	Same as base case, except changed (i) and (ii)	n_i_ ∼U(30, 100) for trials 1 and 2, n_i_ ∼U(900, 1000) for trials 3 to 5
**B2‐A2**	Same as base case, except changed (i) and (ii)	n_i_ ∼U(30, 100) for trials 1 to 10, n_i_ ∼U(900, 1000) for trials 11 to 20
**B3**	Same as base case, except changed (ii)	n_i_ ∼U(30, 100)
**C1**	Same as base case, except changed (vii)	Halving τ_β_^2^ to 117
**C2**	Same as base case, except changed (vii)	Doubling τ_β_^2^ to 468
**D1**	Same as base case, except changed (v)	Halving τ ^2^ to 3.9
**D2**	Same as base case, except changed (v)	Doubling τ ^2^ to 15.6

*Each scenario was repeated under the following data generating mechanisms: (1) random treatment effect with a normally distributed intercept, (2) random treatment effect with a 220*beta(15, 3) distribution for the intercept (except scenarios C1 and C2), and (3) common treatment effect with a normally distributed intercept (except scenarios D1 and D2). Abbreviations: K = number of trials, n_i_ = number of participants in trial i, θ = summary treatment effect, τ ^2^ = between trial variation in summary treatment effect, β = mean response in control group, τ_β_^2^ = between trial variation in mean response in control group, σ ^2^ = residual variance, U (a, b) = uniform distribution over the interval (a, b).

We varied the number of trials (scenarios A1 and A2), so that K = 5, 10, and 20 were considered, which cover the typical sizes of IPD meta‐analyses in our experience. We also considered trials with differing sample sizes within an IPD, so that n_i_ (number of participants within trial i) was drawn from a uniform distribution, n_i_ ∼U(a, b). Fixing a = 30, b = 1000 (scenario B1) allowed for mixed sample sizes, and having 5 trials with a = 30, b = 100 and 5 with a = 900, b = 1000 within an IPD (scenario B2) tested the effect of a mix of small and large sample sizes only. Lastly, fixing a = 30, b = 100 tested the effect of having only small trials (scenario B3).

We also tested the combined effect of varying the number of trials and number of participants per trial simultaneously (scenarios B1‐A1, B1‐A2, B2‐A1, B2‐A2), and we tested the effects of adjusting the magnitude of the intercept or treatment effect heterogeneity (scenarios C1, C2, D1, D2).

Scenarios 15 to 38 replicate the first 14 scenarios where possible, for modifications to the base case data generating mechanism. First, to test the robustness of the normality of the intercept assumption in the random intercept model, we altered the final step of the data generating mechanism in Equation (8), so that the final outcome was calculated by

(9) $$\begin{array}{cc} Y_{\mathrm{Fij}} & =\beta_{i}+\left(\theta +u_{2i}\right){\text{treat}}_{\mathrm{ij}}+e_{\mathrm{ij}}\\ & \;\beta_{i}∼\left(\text{Beta}\left(15,3\right)\right)\times 220\\ & \;u_{2i}∼N\left(0,\tau^{2}\right)\\ & \;e_{\mathrm{ij}}∼N\left(0,\sigma^{2}\right). \end{array}$$

Therefore, the intercept term β_i_ was now derived from a beta distribution with shape parameters of 15 and 3, which represent a negatively skewed distribution that was then scaled by 220 to give sensible values for systolic blood pressure (the outcome upon which the hypothetical data is based). An example density plot of this beta distribution for modeling the intercept term is shown in Web Figure A.1.

Secondly, we also considered a data generating mechanism with a common (fixed) treatment effect (ie, τ ^2^ = 0). Here, the fitted stratified and random intercept models were also modified to have a common treatment effect.

### 3.2 Results

**Figure 2:**
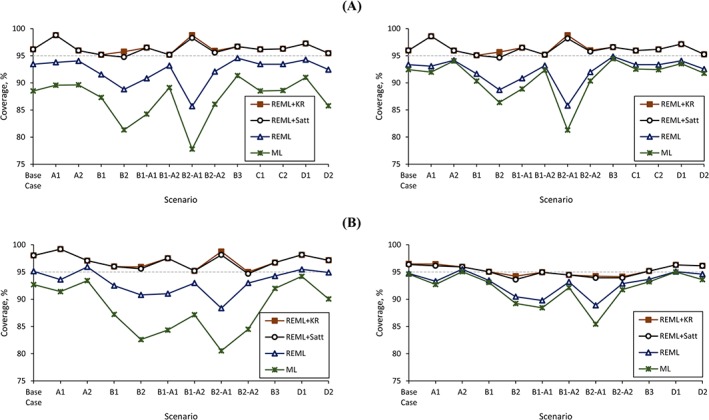
Percentage coverage of the summary treatment effect estimate ( $\hat{\theta })$ under different scenarios for the random treatment effect with normal (Figure 2A) and beta distributions (Figure 2B) for the intercept data generating mechanisms, for stratified (left) and random (right) intercept models, under each of the estimation and CI derivation options considered. Options: ML, maximum likelihood estimation with standard confidence interval (CI) derivation; REML, restricted maximum likelihood estimation with standard CI derivation; REML+KR, REML estimation with Kenward‐Roger CI derivation; REML+Satt, REML estimation with Satterthwaite CI derivation [Colour figure can be viewed at http://wileyonlinelibrary.com]

Simulation results are shown in Tables 2 and 3, covering most of the scenarios under the normal and beta distribution intercept data generating mechanisms, across all options for specifying and estimating the intercept. These tables show the mean percentage bias of the summary treatment effect estimate $\left(\hat{\theta }\right)$ (Table 2) and the median percentage bias in its heterogeneity $({\hat{\tau }}^{2}$) (Table 3). Figure 2 graphically depicts the percentage coverage of the summary treatment effect estimate $\left(\hat{\theta }\right)$.

**Table 2:** Mean percentage bias of the summary treatment effect estimate ( $\hat{\theta })$ under different scenarios, for the random treatment effect with normal and beta distributions for the intercept data generating mechanisms. Results shown separately for stratified (1) and random (2) intercept models, under each of the different estimation options considered

	Mean Percentage Bias of $\hat{\theta }$										
Intercept	Normal Distribution						Beta Distribution				
Generating											
Mechanism											
Method for	Stratified Intercept			Random Intercept			Stratified Intercept			Random Intercept	
Modeling											
Intercept											
Estimation	ML	REML		ML	REML		ML	REML		ML	REML
Scenario *											
**Base case**	−0.01	0.00		−0.01	−0.01		0.34	0.31		0.33	0.29
**A1**	−0.90	−0.90		−0.90	−0.90		−0.02	0.13		−0.06	0.10
**A2**	0.15	0.18		0.16	0.18		−0.48	−0.41		−0.47	−0.40
**B1**	0.67	0.58		0.68	0.58		−0.57	−0.63		−0.58	−0.63
**B2**	−0.47	−0.59		−0.47	−0.56		0.29	0.27		0.33	0.28
**B1‐A1**	0.54	0.53		0.53	0.53		−0.14	−0.27		−0.11	−0.24
**B1‐A2**	−0.10	−0.11		−0.10	−0.11		0.08	−0.01		0.10	0.01
**B2‐A1**	−0.41	−0.43		−0.37	−0.41		0.46	0.52		0.05	−0.17
**B2‐A2**	−0.45	−0.41		−0.44	−0.42		−0.45	−0.37		−0.37	−0.34
**B3**	0.19	0.24		0.21	0.25		1.36	1.34		1.20	1.20
**C1**	−0.03	−0.02		−0.03	−0.02		n/a	n/a		n/a	n/a
**C2**	−0.01	0.07		−0.01	0.07		n/a	n/a		n/a	n/a
**D1**	−0.10	−0.13		−0.10	−0.13		0.26	0.34		0.24	0.32
**D2**	0.13	0.12		0.13	0.12		0.46	0.49		0.45	0.46

* See Table 1 for full data generation details relating to each scenario. True value for θ is −9.66. n/a = not applicable, since there is no τ_β_^2^ to vary when a beta distribution is used for the intercept data generating mechanism. Options: ML, maximum likelihood estimation; REML, restricted maximum likelihood estimation.

**Table 3:** Median percentage bias of the between‐trial variance of treatment effects ( ${\hat{\tau }}^{2})$, under different scenarios for the random treatment effect with normal and beta distributions for the intercept data generating mechanisms. Results shown separately for stratified and random intercept models, under each of the estimation options considered

		Median Percentage Bias of ${\hat{\tau }}^{2}$										
Intercept		Normal Distribution						Beta Distribution				
Generating												
Mechanism												
Method for		Stratified Intercept			Random Intercept			Stratified Intercept			Random Intercept	
Modeling												
Intercept												
Estimation		ML	REML		ML	REML		ML	REML		ML	REML
Scenario *												
**Base Case**		−100.00	−16.86		−41.50	−15.85		−100.00	−14.36		−56.17	−32.86
**A1**		−100.00	−36.88		−80.33	−33.10		−100.00	−73.62		−100.00	−80.15
**A2**		−100.00	−8.59		−20.86	−7.74		−100.00	−13.96		−39.78	−25.06
**B1**		−49.64	−10.74		−22.94	−10.09		−100.00	−14.23		−28.91	−16.39
**B2**		−56.93	−18.03		−35.11	−17.84		−72.90	−17.92		−35.95	−19.81
**B1‐A1**		−77.28	−19.57		−42.62	−18.45		−100.00	−27.27		−54.23	−30.91
**B1‐A2**		−40.64	−5.83		−13.08	−6.31		−88.22	−9.50		−19.59	−13.53
**B2‐A1**		−72.73	−28.35		−56.23	−28.10		−100.00	−34.30		−64.33	−32.61
**B2‐A2**		−36.66	−4.98		−14.36	−5.39		−50.99	−4.84		−12.86	−5.35
**B3**		−100.00	−28.68		−61.86	−24.74		−100.00	−17.42		−81.05	−48.75
**C1**		−100.00	−16.72		−39.54	−14.38		n/a	n/a		n/a	n/a
**C2**		−100.00	−16.86		−40.56	−15.94		n/a	n/a		n/a	n/a
**D1**		−100.00	−19.20		−66.97	−24.63		−100.00	−33.67		−99.98	−62.07
**D2**		−100.00	−11.65		−30.04	−11.79		−100.00	−10.50		−37.84	−22.19

* See Table 1 for full data generation details relating to each scenario. True value for τ^2^ is 7.79, except scenarios D1 and D2 where τ^2^ is equal to 3.9 and 15.6, respectively. n/a = not applicable, since there is no τ_β_^2^ to vary when a beta distribution is used for the intercept data generating mechanism. Options: ML, maximum likelihood estimation; REML, restricted maximum likelihood estimation.

We focus on the results when assuming a random treatment effect. Further results assuming a common treatment effect data generating mechanism and for additional performance measures (percentage convergence of models, numerical percentage coverage of the summary treatment effect estimate, average run time of simulations, and empirical SE and MSE of the summary treatment effect estimate) are shown in the supplementary material (Web Appendices B and C, respectively). In the following, we summarize the key findings.

#### 3.2.1 Convergence of models

Under a random treatment effect data generating mechanism, the proportion of models that converged was consistently high, with a minimum convergence of 94.3% across all situations (Web Table C.I).

Note that all other performance measures to follow are estimated conditional on model convergence.

#### 3.2.2 Bias of summary treatment effect estimate

Generally, there were negligible differences in mean percentage bias of $\hat{\theta }$ between ML and REML estimation options for either model (stratified or random intercept), under any given scenario and data generating mechanism (Table 2 and Web Table B.I). Nor were there any important differences in the mean percentage bias of $\hat{\theta }$ between the stratified model and random intercept model. Furthermore, mean bias was close to zero in all situations and only reached a maximum absolute percentage of 1.36%.

#### 3.2.3 Bias of estimated between‐trial variance of treatment effects

For either model (stratified or random intercept), under any given scenario and data generating mechanism, using ML always produced more downwardly biased estimates than REML (Table 3), as expected.(14, 15, 16, 17, 18) For example, for the base case scenario with the random intercept model, under the normal intercept data generating mechanism, the median percentage bias using REML estimation was −15.9% compared to −41.5% using ML estimation. The bias was worse when using a stratified intercept model (due to the extra number of parameters to estimate), as ML estimation often produced a downward median bias of 100%.

When using REML estimation, there were generally only small differences between random and stratified intercept models in terms of bias of the between‐trial variance of treatment effects; however, while better than ML, downward bias was not removed entirely with REML. Furthermore, the overall size of the bias was typically greater in the beta distribution intercept case than in the normal distribution intercept case, regardless of which model was used.

#### 3.2.4 Empirical SE and MSE of summary treatment effect estimate

There were negligible differences in empirical SE or MSE of $\hat{\theta }$ between the two models (stratified or random intercept), under any given scenario and data generating mechanism (Web Tables C.VIII to C.X).

#### 3.2.5 Coverage of summary treatment effect estimate

There were marked differences observed in the coverage of $\hat{\theta }$ across the different estimation approaches (ML or REML) and CI derivations (standard, KR, or Satterthwaite), as now explained.

*(i) Under a normal distribution intercept generating mechanism*

We consider first the normal distribution intercept generating mechanism (Figure 2A and Web Table C.II). Across both models and all scenarios, ML with standard CI (ML + standard) derivation always exhibited under‐coverage compared to the other options (REML+standard, REML+KR, REML+Satterthwaite). For example, for scenario B2 using the stratified intercept model, the percentage coverage using ML + standard was 81.3% compared to 88.8%, 95.8%, and 94.8% using REML+standard, REML+KR and REML+Satterthwaite, respectively. The random intercept model always performed better with respect to coverage under ML than the stratified intercept model under ML, likely due to the reduction in the number of parameters that needed estimation. For example, when considering only small trials (scenario B3), percentage coverage improved from 91.3% to 94.5% (close to the nominal 95% level), when comparing the stratified to a random intercept model with ML estimation.

Using REML substantially improved on the coverage obtained from ML and removed any important differences between the stratified and random intercept models. However, for either model (stratified or random intercept), REML+standard still had important under‐coverage in some scenarios. For example, in scenario B2‐A1, a percentage coverage of 85.7% and 85.8% was observed, under a stratified and random intercept model, respectively.

The REML+KR approach generally improved on the coverage compared to REML+standard, again with no important differences observed between the stratified and random intercept models. Percentage coverage ranged from 95.1 to 98.8% using REML+KR, while the percentage coverage ranged from 85.7% to 94.9% using REML+standard. The improvement gained by using REML+KR was especially important for scenarios that involved at least 10 trials and a large variation in sample sizes (B1, B2, B1‐A2, B2‐A2). For example, for scenario B2 (five small and five large sample sized trials, with average sample size 66 and 949 in the small and large trials, respectively), percentage coverage from the stratified intercept model was 88.8%, using REML+standard, but 95.8% using REML+KR.

Using REML+Satterthwaite gave very similar results to REML+KR. Occasionally, there was some over‐coverage using REML+KR or REML+Satterthwaite, particularly when using a low number of trials (*K* = 5). For example, coverage was close to 99% (regardless of which model was used), in a setting of *K* = 5 trials with an equal number of participants per trial (scenario A1; *n*_*i*_ = 100), and in a setting of *K* = 5 trials with some small‐sized and some large‐sized trials (scenario B2‐A1; 2 small trials where *n*_*i*_ *∼*U(30, 100), and 3 large trials where *n*_*i*_ *∼*U(900, 1000)).

*(ii) Under a beta distribution intercept generating mechanism*

For the beta distribution intercept generating mechanism (Figure 2B and Web Table C.III), using REML+standard again gave better coverage than using ML, and using REML+KR or REML+Satterthwaite generally further improved upon this coverage (ie, moved it closer to 95%), especially with scenarios concerning at least 10 trials that had a large variation in sample sizes.

As before, under ML estimation, the random intercept model showed better estimates of between‐trial variance and improved coverage (closer to 95%) than the stratified intercept model. However, differences between the two models were generally small for estimation under REML (with or without a 95% CI correction).

#### 3.2.6 Common treatment effect data generating mechanism

Results based on a common (fixed) treatment effect data generating mechanism are shown in Web Appendix B. All fitted models assumed a common treatment effect and converged every time (ie, 100% convergence), and there was negligible difference in mean percentage bias of $\hat{\theta }$ between ML and REML estimation options for either model (stratified or random intercept), or between either model (Web Table B.1). The percentage coverage results were stable across all comparisons, ranging from 93.8 to 96.0%, with negligible differences between the various models and estimation options (Web Figure B.1).

#### 3.2.7 Key findings

**Figure 3:**
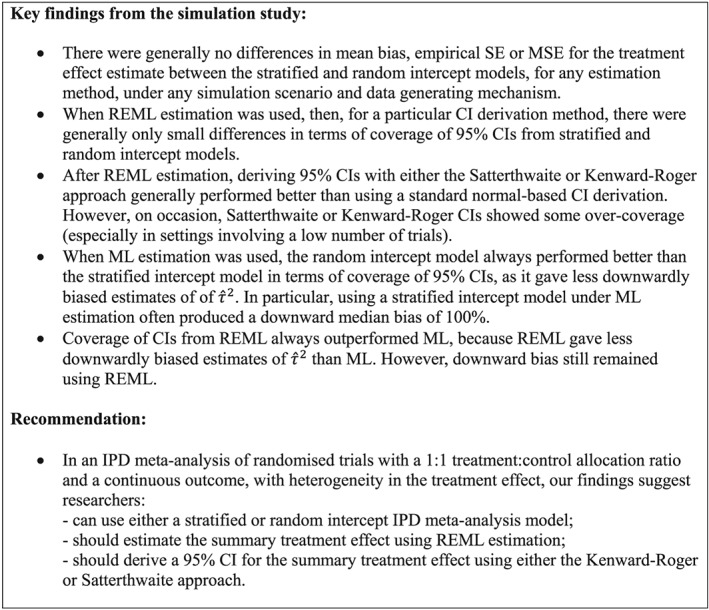
Key simulation findings and recommendation for estimating a summary treatment effect based on a one‐stage individual participant data (IPD) meta‐analysis of randomized trials with a 1:1 treatment:control allocation ratio and a continuous outcome, with between‐study heterogeneity in the treatment effect. CI, confidence interval; ML, maximum likelihood estimation; MSE, mean square error; REML, restricted maximum likelihood; SE, standard error

A summary of the key findings from this simulation study for settings with between‐trial heterogeneity in the treatment effect is given in Figure 3.

## 4 ILLUSTRATION OF METHODS AND KEY FINDINGS IN A REAL EXAMPLE

The International Weight Management in Pregnancy (i‐WIP) Collaborative Group dataset includes IPD from 36 trials (12,447 women), collected for a Health Technology Assessment report in 2017.^24^ The authors investigated the association between diet and lifestyle interventions to prevent weight gain in pregnancy and several other primary outcomes. Here, we present IPD meta‐analysis results using the i‐WiP dataset, for illustration purposes only, to demonstrate the key findings from our simulation study. We include only trials that collected follow‐up outcome values for weight in pregnancy and apply a one‐stage IPD model for this continuous outcome, with model assumptions in line with our simulation study analysis. However, while we did not generate any baseline imbalances in our simulation data, baseline weight imbalance was present in some trials from the i‐WIP data. To remove this imbalance, we apply a baseline adjustment in our models, as is recommended.^21^

Table 4 shows IPD meta‐analysis results for a random sample of 5 and 10 trials that investigated exercise interventions and for 20 trials (using all 15 exercise trials, plus 5 additional trials that investigated mixed interventions).

**Table 4:** Results from baseline weight adjusted individual participant data meta‐analysis of i‐WIP data: summary treatment effect estimate ( $\hat{\theta })$ with 95% confidence interval and between‐trial variance of treatment effects estimate ( ${\hat{\tau }}^{2})$. From meta‐analysis with different numbers of trials (K = 5, 10, or 20), and assuming a random treatment effect and a common residual variance throughout

	$\hat{\theta }$ (95**%** CI); ${\hat{\tau }}^{2}$								
Method for Modeling	Stratified					Random			
Intercept	Intercept					Intercept			
Estimation	ML	REML	REML+KR	REML+Satt		ML	REML	REML+KR	REML+Satt
***Number of trials***									
**5**	−1.172	−1.172	−1.172	−1.172		−1.170	−1.171	−1.171	−1.171
	(−1.811, −0.534);	(−1.815, −0.530);	(−3.114, 0.770);	(−2.712, 0.367);		(−1.811, −0.529);	(−1.813, −0.528);	(−3.072, 0.731);	(−2.681, 0.340);
	8.58E−17	3.94E−15	3.94E−15	3.94E−15		2.95E−14	4.51E−12	4.51E−12	4.51E−12
**10**	−0.972	−0.972	−0.972	−0.972		−0.972	−0.972	−0.972	−0.972
	(−1.479, −0.465);	(−1.482, −0.462);	(−1.740, −0.204);	(−1.653, −0.291);		(−1.481, −0.462);	(−1.482, −0.462);	(−1.731, −0.212);	(−1.646, −0.298);
	1.97E−16	2.58E−12	2.58E−12	2.58E−12		9.52E−11	5.94E−16	5.94E−16	5.94E−16
**20**	−0.821	−0.820	−0.820	−0.820		−0.830	−0.830	−0.830	−0.830
	(−1.102, −0.540);	(−1.243, −0.396);	(−1.298, −0.342);	(−1.286, −0.354);		(−1.217, −0.442);	(−1.235, −0.426);	(−1.290, −0.370);	(−1.276, −0.384);
	1.11E−14	0.317	0.317	0.317		0.210	0.258	0.258	0.258

CI = confidence interval Options: ML, maximum likelihood estimation with standard CI derivation; REML, restricted maximum likelihood estimation with standard CI derivation; REML+KR, REML estimation with Kenward‐Roger CI derivation; REML+Satt, REML estimation with Satterthwaite CI derivation.

These results are in agreement with the key findings observed in Section 3.2 and summarized in Figure 3. Firstly, the magnitude of summary treatment effect estimate was similar throughout, irrespective of model used or estimation method. Secondly, with ML estimation, the stratified intercept model gave narrower 95% CIs and smaller estimates of the between trial variance than the random intercept model, especially with *K* = 20 trials. Thirdly, using REML overcame this discrepancy, with now very similar results between the random and stratified intercept models; in addition, the 95% CIs were wider when using REML, due to the larger estimates of the between trial variance. Fourthly, applying a KR or Satterthwaite correction in addition to REML further widened the 95% CIs. Finally, although 95% CIs were slightly wider when using a KR correction instead of Satterthwaite, results were generally similar from these two corrections, especially with *K* = 20 trials.

## 5 DISCUSSION

### 5.1 Key findings

In summary, we have conducted an extensive simulation study to examine the estimation of a summary treatment effect using a one‐stage IPD meta‐analysis model for a continuous outcome. Specifically, we examined different options for specifying the trial‐specific intercepts and compared different options for parameter estimation and CI derivation. Fourteen different scenarios were tested (varying the number of trials, number of participants per trial, and heterogeneity of parameters), for each of three different data generating mechanisms (encompassing a common and random treatment effect with a normally distributed intercept, as well as a beta distributed intercept and random treatment effect). All scenarios assumed a 1:1 treatment:control allocation ratio, and a data generating mechanism that was based on a random intercept model; hence, our conclusions are restricted to this context.

Our key findings, for settings with heterogeneity in treatment effect, were illustrated using a real example, and these are summarized in Figure 3. Firstly, the results suggest that, as long as the same estimation method is used, there are no important differences between the stratified and random intercept models in terms of bias, empirical SE or MSE for the summary treatment effect estimate. Indeed, the mean bias in $\hat{\theta }$ was close to zero throughout, which is perhaps expected given the statistical theory underpinning linear mixed models. Furthermore, when using REML (with or without a CI correction), there were generally no important differences in coverage performance between the stratified and random intercept models. Interestingly, the random intercept model (which assumes normality of the intercept) performed well even when the trial intercepts were drawn from a highly asymmetric beta distribution. Kahan and Morris^25^ also found that misspecifying the random intercept distribution of random effects models did not impact treatment effect results.

Secondly, the KR and Satterthwaite corrections generally performed similarly in terms of improving the coverage and were especially effective for scenarios involving at least 10 trials with a mix of small and large sample sizes, but also considerably increased mean run time in these instances (see Web Tables C.V to C.VII). The Satterthwaite correction always had a similar or quicker average run time than KR (sometimes by more than eight times). One could surmise from the similarity in coverage performance that the main impact of both corrections is in the use of a t‐distribution to derive CIs and that the KR adjusted variance of the summary estimate has relatively less impact.

Thirdly, when using ML estimation, the random intercept model always showed better or comparable coverage to the stratified intercept model (closer to 95%). This is likely due to the random intercept model having a reduced number of parameters, and thus improved ML estimation of the between‐trial variance. A similar finding was also recently shown by Jackson et al^26^ for one‐stage meta‐analysis models for a binary outcome. Nevertheless, even the random intercept model produced downwardly biased estimates of the between‐trial variance using ML and low coverage. Using REML is therefore important, to improve on this coverage. Indeed, coverage is more consistently near 95% when using REML with either a KR or Satterthwaite correction. However, on some occasions (particularly, when there are a low number of trials), the KR and Satterthwaite corrections lead to over‐coverage. This is similar to the Hartung‐Knapp Sidik‐Jonkman correction to 95% CIs following a two‐stage analysis,(27, 28) which generally gives a more suitable coverage than a standard 95% CI, although on occasion is overly conservative.^29^

If there is genuinely no heterogeneity in treatment effect across trials, however, our findings suggest that there are generally no differences in mean bias, empirical SE, MSE, or coverage for the treatment effect between the stratified and random intercept models, for any estimation method, CI derivation approach, and under any simulation scenario. However, in our experience, situations of completely homogeneous treatment effects are unlikely.

*Unreported simulations*

Following recent work by Morris et al^5^ and Jackson et al,^26^ which considered an alternative coding for the binary treatment group variable (+0.5/−0.5 for treatment/control groups, respectively), we also tested this treatment group coding for our REML estimation simulation results but found only small differences in performance results compared to the 1/0 coding. Hence, we did not present the results here. We also tested using stratified (instead of common) residual variances for both the data generating mechanisms and models fitted. Again, no difference in performance of the summary treatment effect estimate was observed, suggesting that the IPD model may be robust to the (mis) specification of the residual variances. Morris et al^5^ also found that assuming common or distinct residual variances, in a common treatment effect IPD meta‐analysis setting, has very little impact on the precision of the summary effect when the number of patients per trial is over 25. In general, from a point of principle, we recommend a separate residual variance for each trial, but in situations where this has convergence problems, a common residual variance would seem apt. Further research of this issue would be welcome.

### 5.2 Recommendation

For researchers conducting a one‐stage IPD meta‐analysis of randomized trials with a 1:1 treatment:control allocation ratio and a continuous outcome and aiming to estimate a summary treatment effect that is heterogeneous across trials, we recommend that either a stratified or random intercept model is used, and estimated using REML, ideally followed by a 95% CI derived using either the KR or Satterthwaite approaches. In our simulations, this approach gave close to zero mean bias in the summary treatment effect estimate and coverage generally close to 95%, except in a few situations where there was over‐coverage (particularly, when there were a low number of trials).

Using REML with a KR correction for linear mixed models based on continuous outcomes has already been proposed by some researchers,(30, 31) while literature advocating the merits of the Satterthwaite correction is less common. However, in our simulations, we found that the Satterthwaite correction generally obtains similar results to the KR approach, hence making for an excellent alternative.

### 5.3 Limitations and further research

Throughout this simulation study, we focused solely on synthesizing trials containing a 1:1 treatment:control allocation ratio; hence, an important limitation is that our conclusions may not hold under settings involving other treatment allocation ratios.

In addition, we have focused solely on IPD of continuous outcomes, hence another important limitation is that our conclusions are not necessarily generalizable to other popular outcome types in the meta‐analytical field, such as binary and time‐to‐event outcomes. Binary outcomes, for example, are more complex to deal with than continuous outcomes, as a logistic mixed effects model is nonlinear, and hence, the corresponding maximum likelihood function has no closed form. Jackson et al^26^ recently investigated the use of ML estimation and found that a stratified intercept model leads to substantial downward bias in between‐trial variance estimates and under‐coverage of CIs for the summary result, which increases as the number of trials (and thus parameters) increases. Interestingly, the issue was resolved when using a + 0.5/−0.5 coding for the treatment variable, rather than a 1/0 coding, or when placing random effects on the trial intercept.^26^ McNeish^32^ investigated logistic mixed models by either retaining the nonlinearity of the model and making an approximation for the likelihood function or linearly approximating the model to give the likelihood function a closed form (pseudo‐likelihood approach). The latter option was shown to be favorable (under the specific conditions of the study), by use of a residual penalized quasi‐likelihood with a KR correction.

While our simulation study did consider an extensive range of scenarios—we varied the number of trials, number of participants per trial, and heterogeneity of parameters—we recognize that our conclusions were based on a final score model that did not adjust for baseline outcome value. Often, the ANCOVA model should be used, as in our applied example, because there will be baseline imbalances in practice. However, as baseline values did not vary across individuals in our simulation study, using a final score analysis model rather than ANCOVA was appropriate. When using one‐stage ANCOVA IPD models, an additional issue is using stratified adjustment terms or placing a random effect on the adjustment term. Based on our study findings, we expect that, with REML, either approach should be suitable.

We also assumed independence of the two random effects (ie, a covariance of zero) when assuming random intercept and random treatment effects, both in the data generating mechanism and when fitting the corresponding model. Their correlation could be taken into account if deemed sensible^13^; however, we did not consider this alternative assumption in our simulation study, largely due to the added complexity and difficulty in estimating the correlation parameter in practice with few trials. Importantly, it is perhaps likely that the effect of treatment could be correlated with the control group outcome, and therefore, the most appropriate assumption needs further consideration.

Another limitation is that we did not consider prediction intervals. These allow us to make predictive inferences of the potential treatment effect in a single setting of application.^33^ Some researchers argue that prediction intervals offer a more appropriate summary of trial findings than CIs of the average effect.^34^ However, Partlett and Riley^18^ showed in a two‐stage IPD meta‐analysis setting that there was considerable under‐coverage of prediction intervals in some situations. For example, under‐coverage was observed in settings involving a low heterogeneity or with varied trial sample sizes and was not improved upon by increasing the number of trials or using CI corrections such as KR. Hence, we did not consider it useful to consider prediction intervals in our study.

Finally, we could have considered a Bayesian approach to our simulation study, which is an alternative to frequentist methods, and a natural way to account for all parameter uncertainty, to make predictions and to derive (joint) probabilistic statements regarding parameters of interest. However, we deemed this extension to be beyond the scope of this paper. Yet, if a Bayesian approach is to be used in practice, Bayesians still need to choose between random or stratified intercept one‐stage IPD models, which is something that our work can help with.

### 5.4 Conclusions

In an IPD meta‐analysis of trials with a 1:1 treatment:control allocation ratio and a continuous outcome, aiming to estimate a summary treatment effect that is heterogeneous across trials, our findings suggest that researchers use either a stratified or random intercept model with REML estimation and ideally derive 95% CIs using either the KR or Satterthwaite approach. Further work is needed to improve upon coverage in a few situations where the KR and Satterthwaite intervals are overly conservative. Such situations include when there are a low number of trials; these are also situations where corrections to CIs in a two‐stage IPD meta‐analysis are overly conservative.^18^

## AUTHOR CONTRIBUTIONS

Richard D. Riley and Danielle L. Burke developed the research idea. Amardeep Legha undertook all the simulation analyses under the supervision of Richard D. Riley and Danielle L. Burke and feedback from Joie Ensor, Kym I. E. Snell, and Tim P. Morris. Danielle L. Burke performed analyses for the real example. Amardeep Legha drafted the paper and revised following comments and revisions from Danielle L. Burke, Richard D. Riley, Joie Ensor, Kym I. E. Snell, and Tim P. Morris.

## Supporting information

SIM_7930‐Supp Material (3 Appendices)_10.07.18.docx
