# Supplementary material for: Individual participant data meta‐analysis of continuous outcomes: A comparison of approaches for specifying and estimating one‐stage models
Source: Stat Med. 2018 Aug 13;37(29):4404–20. doi: 10.1002/sim.7930 (PMC6283045; doi:10.1002/sim.7930)
Supplement: Supplementary file 1 — SIM_7930‐Supp Material (3 Appendices)_10.07.18.docx [file SIM-37-4404-s001.docx]

**Web Appendix: Supplementary Material**

**Web Appendix A: Definitions of summary performance measures, and beta distribution density plot**

The mean bias of $\hat{\theta}$ is calculated as:

$Mean bias of \hat{\theta}=mean\left[ \hat{\theta} \right]-\theta$, (1)

that is, the difference in the average estimate of $\hat{\theta}$ (from up to 1000 simulations) and the true value of 𝜃.

Hence the mean percentage bias of $\hat{\theta}$ is defined as:

$Mean percentage bias of \hat{\theta}=\left. \left( \frac{mean\left[ \hat{\theta} \right]-\theta}{\theta} \right) \right.\text{x}100\%$ (2)

To summarise *τ^2^*, median percentage bias was used due to its skewed nature; calculated using the same approach as equation 2.

The precision of an estimate is also important to consider. The empirical SE of $\hat{\theta}$ is defined as:

$Empirical SE of \hat{\theta}=\sqrt{Var(\hat{\theta})}$, (3)

which assesses the spread of the (up to 1000) estimates of *θ*. The MSE then incorporates a measure of both accuracy (bias) and variability (empirical SE) for the estimator:

$MSE of \hat{\theta}={(Mean bias of \hat{\theta})}^{2}+{(Empirical SE of \hat{\theta})}^{2}$ (4)

The coverage of a confidence interval of $\hat{\theta}$ is the proportion of times the (up to) 1000 simulated confidence intervals of $\hat{\theta}$contain the true value of 𝜃. If the true coverage was 95%, with 1000 simulations, we would expect (with 95% confidence) the observed coverage to be between 93.6% and 96.4% due to Monte Carlo error.

We define percentage convergence as:

$Convergence \%=\left( \frac{Number of simulations converged within 100 iterations}{Total number of simulation replications} \right)\text{x}100\%$ (5)

Run-time is defined as the total time taken (in seconds) from the start of model fit to the end of post estimation (i.e. 95% CI derivation). High convergence (close to 100%) and short average run-time (close to zero seconds) are desirable properties, conditional on the estimation properties (e.g. bias) themselves being appropriate.


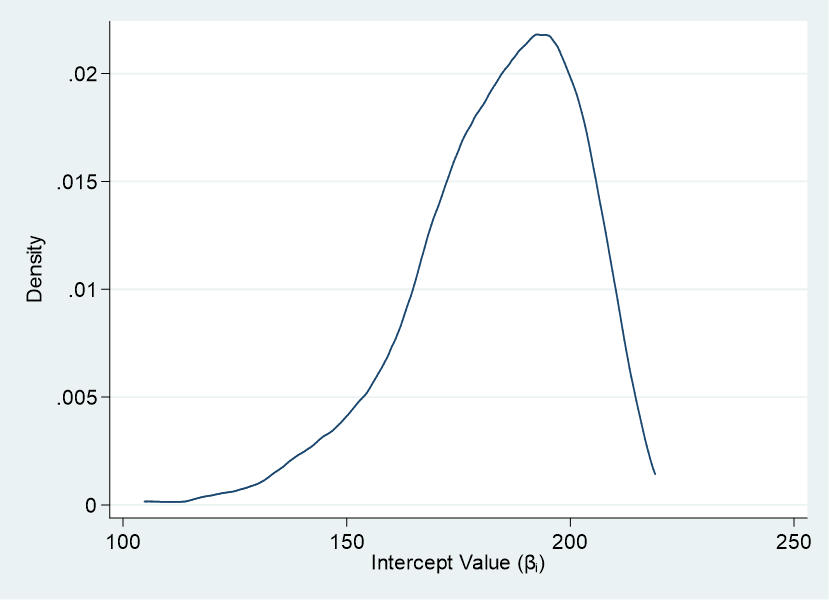


**Web Figure A.1.** Example density plot of intercept term, *β_i_*, derived from a 220*beta(15,3) distribution simulated dataset of 1000 observations

**Web Appendix B: Common (fixed) treatment effect data generating mechanism main simulation results**

**Web Table B.I.** Mean percentage bias of the summary treatment effect estimate ($\hat{\theta})$, under different scenarios for the common treatment effect data generating mechanism. Results shown separately for stratified and random intercept models, under each of the different estimation options considered.

|  |  | **Mean percentage bias of** $\hat{\boldsymbol{\theta}}$ | | | | |
| --- | --- | --- | --- | --- | --- | --- |
| **Method for modelling intercept** |  | **Stratified  intercept** | |  | **Random  intercept** | |
| **Estimation** |  | **ML** | **REML** |  | **ML** | **REML** |
| *Scenario** |  |  |  |  |  |  |
| **Base Case** |  | -0.52 | -0.52 |  | -0.52 | -0.52 |
| **A1** |  | 0.86 | 0.86 |  | 0.87 | 0.87 |
| **A2** |  | 0.18 | 0.18 |  | 0.17 | 0.17 |
| **B1** |  | 0.02 | 0.02 |  | 0.02 | 0.02 |
| **B2** |  | 0.06 | 0.06 |  | 0.06 | 0.06 |
| **B1-A1** |  | 0.11 | 0.11 |  | 0.10 | 0.10 |
| **B1-A2** |  | 0.02 | 0.02 |  | 0.02 | 0.02 |
| **B2-A1** |  | -0.45 | -0.45 |  | -0.45 | -0.45 |
| **B2-A2** |  | 0.04 | 0.04 |  | 0.04 | 0.04 |
| **B3** |  | -0.06 | -0.06 |  | -0.06 | -0.06 |
| **C1** |  | -0.52 | -0.52 |  | -0.52 | -0.52 |
| **C2** |  | -0.52 | -0.52 |  | -0.52 | -0.52 |

*** See Table I for full data generation details relating to each scenario. True value of *θ* is -9.66.

Options: ML, maximum likelihood estimation; REML, restricted maximum likelihood estimation.


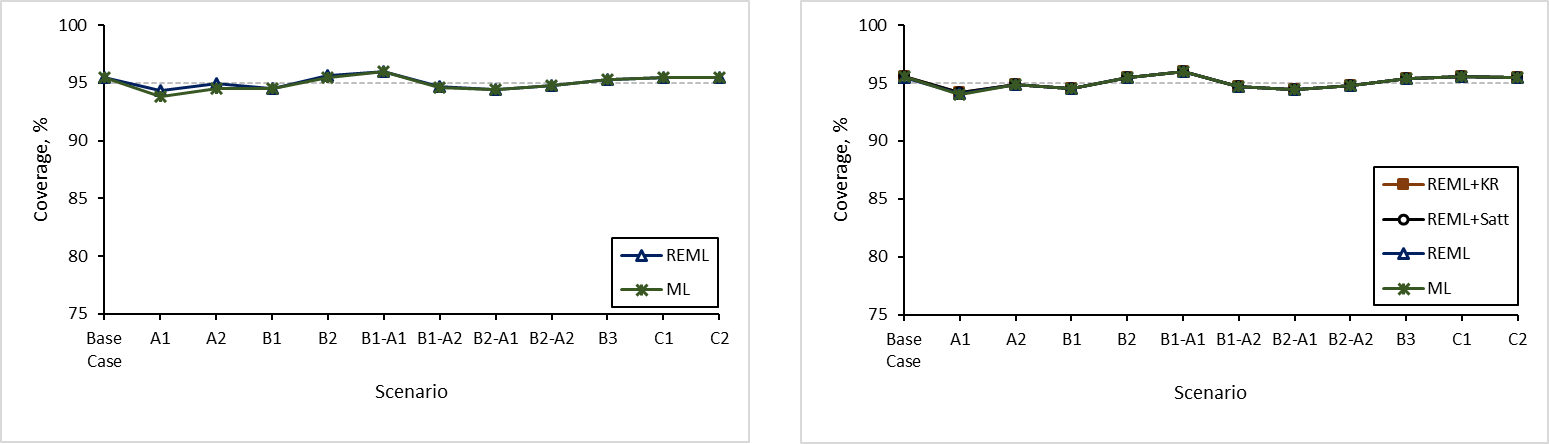


**Web Figure B.1.** Percentage coverage of the summary treatment effect estimate ($\hat{\theta})$, under different scenarios for the common treatment effect data generating mechanism, for a stratified (left) and a random intercept model (right), under each of the different estimation and CI derivation options considered.

Options: ML, maximum likelihood estimation with standard confidence interval (CI) derivation; REML, restricted maximum likelihood estimation with standard CI derivation; REML+KR, REML estimation with Kenward-Roger CI derivation; REML+Satt, REML estimation with Satterthwaite CI derivation. (Note: KR and Satterthwaite correction options not available for stratified intercept model as model does not include any random effects).

**Web Appendix C: Other simulation results (convergence, coverage, run times, empirical SE, and MSE)**

**Web Table C.I.** Percentage convergence of models under different scenarios for the random treatment effect with normal and beta distributions for the intercept data generating mechanisms. Results shown separately for stratified (1) and random intercept models (2), under each of the different estimation options considered.

|  |  | **Percentage convergence** | | | | | | | | | | |
| --- | --- | --- | --- | --- | --- | --- | --- | --- | --- | --- | --- | --- |
| **Intercept generating mechanism** |  | **Normal distribution** | | | | |  | **Beta distribution** | | | | |
|  |  |  |  |  |  |  |  |  |  |  |  |  |
| **Method for modelling intercept** |  | **Stratified intercept** | |  | **Random intercept** | |  | **Stratified intercept** | |  | **Random**  **intercept** | |
| **Estimation** |  | **ML** | **REML** |  | **ML** | **REML** |  | **ML** | **REML** |  | **ML** | **REML** |
| *Scenario** |  |  |  |  |  |  |  |  |  |  |  |  |
| **Base Case** |  | 99.9 | 99.8 |  | 99.3 | 99.5 |  | 99.9 | 98.6 |  | 98.4 | 97.9 |
| **A1** |  | 100.0 | 99.7 |  | 99.7 | 100.0 |  | 100.0 | 99.7 |  | 98.8 | 98.8 |
| **A2** |  | 99.9 | 99.3 |  | 99.3 | 99.8 |  | 99.7 | 98.7 |  | 97.4 | 97.2 |
| **B1** |  | 99.3 | 99.7 |  | 99.9 | 99.7 |  | 99.2 | 98.7 |  | 96.1 | 96.0 |
| **B2** |  | 99.4 | 99.5 |  | 99.7 | 99.6 |  | 99.2 | 98.9 |  | 97.3 | 96.6 |
| **B1-A1** |  | 99.7 | 99.6 |  | 99.3 | 99.6 |  | 99.7 | 98.8 |  | 97.9 | 98.0 |
| **B1-A2** |  | 99.5 | 99.8 |  | 99.9 | 99.9 |  | 99.3 | 99.6 |  | 94.8 | 94.3 |
| **B2-A1** |  | 99.8 | 99.6 |  | 99.7 | 99.5 |  | 99.9 | 99.1 |  | 98.2 | 97.9 |
| **B2-A2** |  | 99.9 | 99.7 |  | 99.8 | 99.7 |  | 98.7 | 99.6 |  | 94.8 | 94.3 |
| **B3** |  | 100.0 | 99.6 |  | 99.3 | 99.6 |  | 100.0 | 99.1 |  | 98.5 | 98.5 |
| **C1** |  | 99.9 | 99.8 |  | 99.3 | 99.3 |  | n/a | n/a |  | n/a | n/a |
| **C2** |  | 99.9 | 99.8 |  | 99.2 | 99.5 |  | n/a | n/a |  | n/a | n/a |
| **D1** |  | 100.0 | 98.7 |  | 98.9 | 99.0 |  | 100.0 | 98.9 |  | 97.6 | 98.5 |
| **D2** |  | 99.9 | 99.6 |  | 99.8 | 99.6 |  | 99.9 | 99.2 |  | 98.6 | 99.1 |

*See Table I for full data generation details relating to each scenario.

n/a = not applicable, since there is no *τ_β_ ^2^* to vary when a beta distribution is used for the intercept data generating mechanism.
Options: ML, maximum likelihood estimation; REML, restricted maximum likelihood estimation.

**Web Table C.II.** Percentage coverage of the summary treatment effect estimate ($\hat{\theta})$ under different scenarios for the random treatment effect and normal intercept data generating mechanism. Results shown separately for stratified and random intercept models, under each of the estimation options considered.

|  |  |  | **Percentage coverage of** $\hat{\boldsymbol{\theta}}$ | | | | | | | |
| --- | --- | --- | --- | --- | --- | --- | --- | --- | --- | --- |
| **Method for modelling intercept** |  | **Stratified  intercept** | | | |  | **Random  intercept** | | | |
| **Estimation** |  | **ML** | **REML** | **REML+KR** | **REML+Satt** |  | **ML** | **REML** | **REML+KR** | **REML+Satt** |
| *Scenario** |  |  |  |  |  |  |  |  |  |  |
| **Base Case** |  | 88.51 | 93.45 | 96.17 | 96.17 |  | 92.44 | 93.35 | 95.97 | 95.97 |
| **A1** |  | 89.57 | 93.78 | 98.80 | 98.80 |  | 91.98 | 93.08 | 98.60 | 98.60 |
| **A2** |  | 89.62 | 94.05 | 95.96 | 95.96 |  | 94.05 | 94.15 | 95.96 | 95.96 |
| **B1** |  | 87.31 | 91.56 | 95.18 | 95.18 |  | 90.33 | 91.66 | 95.08 | 95.08 |
| **B2** |  | 81.33 | 88.80 | 95.76 | 94.75 |  | 86.38 | 88.70 | 95.66 | 94.65 |
| **B1-A1** |  | 84.24 | 90.84 | 96.48 | 96.48 |  | 88.89 | 90.84 | 96.48 | 96.48 |
| **B1-A2** |  | 89.13 | 93.18 | 95.19 | 95.19 |  | 92.35 | 93.18 | 95.19 | 95.19 |
| **B2-A1** |  | 77.79 | 85.69 | 98.79 | 98.29 |  | 81.31 | 85.79 | 98.79 | 98.19 |
| **B2-A2** |  | 86.06 | 92.08 | 95.89 | 95.59 |  | 90.37 | 91.98 | 95.99 | 95.79 |
| **B3** |  | 91.34 | 94.56 | 96.68 | 96.68 |  | 94.46 | 94.86 | 96.58 | 96.58 |
| **C1** |  | 88.51 | 93.44 | 96.17 | 96.17 |  | 92.54 | 93.34 | 95.96 | 95.96 |
| **C2** |  | 88.60 | 93.45 | 96.27 | 96.27 |  | 92.43 | 93.35 | 96.17 | 96.17 |
| **D1** |  | 91.00 | 94.27 | 97.24 | 97.24 |  | 93.53 | 94.07 | 97.14 | 97.14 |
| **D2** |  | 85.76 | 92.44 | 95.46 | 95.46 |  | 91.78 | 92.54 | 95.26 | 95.26 |

*See Table I for full data generation details relating to each scenario.

Options: ML, maximum likelihood estimation with standard confidence interval (CI) derivation; REML, restricted maximum likelihood estimation with standard CI derivation; REML+KR, REML estimation with Kenward-Roger CI derivation; REML+Satt, REML estimation with Satterthwaite CI derivation.

**Web Table C.III.** Percentage coverage of the summary treatment effect estimate ($\hat{\theta})$ under different scenarios for the random treatment effect and beta distribution for the intercept data generating mechanism. Results shown separately for stratified and random intercept models, under each of the estimation options considered.

|  |  | **Percentage coverage of** $\hat{\boldsymbol{\theta}}$ | | | | | | | | |
| --- | --- | --- | --- | --- | --- | --- | --- | --- | --- | --- |
| **Method for modelling intercept** |  | **Stratified  intercept** | | | |  | **Random  intercept** | | | |
| **Estimation** |  | **ML** | **REML** | **REML+KR** | **REML+Satt** |  | **ML** | **REML** | **REML+KR** | **REML+Satt** |
| *Scenario** |  |  |  |  |  |  |  |  |  |  |
| **Base Case** |  | 92.68 | 95.13 | 98.03 | 98.03 |  | 94.61 | 94.72 | 96.48 | 96.37 |
| **A1** |  | 91.40 | 93.60 | 99.19 | 99.19 |  | 92.71 | 93.30 | 96.45 | 96.14 |
| **A2** |  | 93.41 | 95.93 | 97.08 | 97.08 |  | 95.06 | 95.52 | 95.93 | 95.93 |
| **B1** |  | 87.21 | 92.50 | 95.99 | 95.99 |  | 93.08 | 93.45 | 95.04 | 95.04 |
| **B2** |  | 82.59 | 90.79 | 95.92 | 95.60 |  | 89.22 | 90.47 | 94.24 | 93.61 |
| **B1-A1** |  | 84.34 | 91.01 | 97.52 | 97.52 |  | 88.43 | 89.77 | 94.94 | 94.94 |
| **B1-A2** |  | 87.15 | 92.97 | 95.21 | 95.21 |  | 92.14 | 93.18 | 94.46 | 94.46 |
| **B2-A1** |  | 80.53 | 88.35 | 98.76 | 98.14 |  | 85.42 | 88.87 | 94.23 | 93.92 |
| **B2-A2** |  | 84.49 | 92.97 | 94.99 | 94.68 |  | 91.76 | 92.86 | 94.14 | 93.93 |
| **B3** |  | 91.98 | 94.26 | 96.72 | 96.72 |  | 93.20 | 93.65 | 95.18 | 95.18 |
| **D1** |  | 94.20 | 95.48 | 98.15 | 98.15 |  | 95.00 | 95.07 | 96.30 | 96.30 |
| **D2** |  | 90.05 | 94.91 | 97.15 | 97.15 |  | 93.60 | 94.61 | 96.13 | 96.13 |

*See Table I for full data generation details relating to each scenario.

Options: ML, maximum likelihood estimation with standard confidence interval (CI) derivation; REML, restricted maximum likelihood estimation with standard CI derivation; REML+KR, REML estimation with Kenward-Roger CI derivation; REML+Satt, REML estimation with Satterthwaite CI derivation.


**Web Table C.IV.** Percentage coverage of the summary treatment effect estimate ($\hat{\theta})$ under different scenarios for the common treatment effect data generating mechanism. Results shown separately for stratified and random intercept models, under each of the estimation options considered.

|  |  | **Percentage coverage of** $\hat{\boldsymbol{\theta}}$ | | | | | | |
| --- | --- | --- | --- | --- | --- | --- | --- | --- |
| **Method for modelling intercept** |  | **Stratified  intercept** | |  | **Random  intercept** | | | |
|  |  |  |  |  |  |  |  |  |
| **Estimation** |  | **ML** | **REML** |  | **ML** | **REML** | **REML+KR** | **REML+Satt** |
| *Scenario** |  |  |  |  |  |  |  |  |
| **Base Case** |  | 95.50 | 95.50 |  | 95.60 | 95.50 | 95.60 | 95.60 |
| **A1** |  | 93.80 | 94.30 |  | 94.00 | 94.10 | 94.20 | 94.20 |
| **A2** |  | 94.50 | 94.90 |  | 94.90 | 94.90 | 94.90 | 94.90 |
| **B1** |  | 94.50 | 94.50 |  | 94.50 | 94.50 | 94.50 | 94.50 |
| **B2** |  | 95.50 | 95.60 |  | 95.50 | 95.50 | 95.50 | 95.50 |
| **B1-A1** |  | 96.00 | 96.00 |  | 96.00 | 96.00 | 96.00 | 96.00 |
| **B1-A2** |  | 94.60 | 94.70 |  | 94.70 | 94.70 | 94.70 | 94.70 |
| **B2-A1** |  | 94.40 | 94.40 |  | 94.40 | 94.40 | 94.40 | 94.40 |
| **B2-A2** |  | 94.80 | 94.80 |  | 94.80 | 94.80 | 94.80 | 94.80 |
| **B3** |  | 95.30 | 95.30 |  | 95.40 | 95.40 | 95.40 | 95.40 |
| **C1** |  | 95.50 | 95.50 |  | 95.60 | 95.60 | 95.60 | 95.60 |
| **C2** |  | 95.50 | 95.50 |  | 95.50 | 95.50 | 95.50 | 95.50 |

*See Table I for full data generation details relating to each scenario.

Options: ML, maximum likelihood estimation with standard confidence interval (CI) derivation; REML, restricted maximum likelihood estimation with standard CI derivation; REML+KR, REML estimation with Kenward-Roger CI derivation; REML+Satt, REML estimation with Satterthwaite CI derivation. (Note: KR and Satterthwaite correction options not available for stratified intercept model as model does not include any random effects).

**Web Table C.V.** Mean run time (in seconds) of simulations, under different scenarios for the normal intercept (random treatment effect) data generating mechanism. Results shown separately for stratified and random intercept models, under each of the different estimation options considered.

|  |  | **Average run time, seconds** | | | | | | | | |
| --- | --- | --- | --- | --- | --- | --- | --- | --- | --- | --- |
| **Method for modelling intercept** |  | **Stratified  intercept** | | | |  | **Random  intercept** | | | |
| **Estimation** |  | **ML** | **REML** | **REML+KR** | **REML+Satt** |  | **ML** | **REML** | **REML+KR** | **REML+Satt** |
| **Scenario*** |  |  |  |  |  |  |  |  |  |  |
| **Base Case** |  | 0.37 | 0.24 | 0.45 | 0.35 |  | 0.21 | 0.20 | 0.57 | 0.37 |
| **A1** |  | 0.21 | 0.16 | 0.25 | 0.21 |  | 0.18 | 0.17 | 0.36 | 0.25 |
| **A2** |  | 1.18 | 0.66 | 1.08 | 0.87 |  | 0.28 | 0.29 | 1.02 | 0.61 |
| **B1** |  | 0.76 | 0.62 | 35.39 | 3.85 |  | 0.46 | 0.44 | 88.55 | 27.47 |
| **B2** |  | 0.84 | 0.66 | 62.66 | 6.50 |  | 0.51 | 0.48 | 163.37 | 57.83 |
| **B1-A1** |  | 0.34 | 0.27 | 16.93 | 1.75 |  | 0.29 | 0.28 | 42.26 | 13.08 |
| **B1-A2** |  | 2.21 | 1.98 | 77.91 | 8.87 |  | 0.83 | 0.83 | 194.21 | 60.18 |
| **B2-A1** |  | 0.39 | 0.32 | 37.13 | 3.75 |  | 0.35 | 0.34 | 96.99 | 36.77 |
| **B2-A2** |  | 2.30 | 2.06 | 170.40 | 13.46 |  | 0.89 | 0.84 | 348.94 | 124.52 |
| **B3** |  | 0.31 | 0.22 | 0.34 | 0.30 |  | 0.19 | 0.19 | 0.37 | 0.29 |
| **C1** |  | 0.36 | 0.28 | 0.43 | 0.35 |  | 0.20 | 0.23 | 0.55 | 0.37 |
| **C2** |  | 0.35 | 0.25 | 0.41 | 0.36 |  | 0.20 | 0.19 | 0.52 | 0.37 |
| **D1** |  | 0.37 | 0.27 | 0.43 | 0.38 |  | 0.22 | 0.21 | 0.53 | 0.38 |
| **D2** |  | 0.30 | 0.20 | 0.37 | 0.32 |  | 0.17 | 0.17 | 0.49 | 0.35 |

*See Table I for full data generation details relating to each scenario.

Options: ML, maximum likelihood estimation with standard confidence interval (CI) derivation; REML, restricted maximum likelihood estimation with standard CI derivation; REML+KR, REML estimation with Kenward-Roger CI derivation; REML+Satt, REML estimation with Satterthwaite CI derivation.

**Web Table C.VI.** Mean run time (in seconds) of simulations, under different scenarios for the beta distribution for the intercept (random treatment effect) data generating mechanism. Results shown separately for stratified and random intercept models, under each of the different estimation options considered.

|  |  | **Average run time, seconds** | | | | | | | | |
| --- | --- | --- | --- | --- | --- | --- | --- | --- | --- | --- |
| **Method for modelling intercept** |  | **Stratified  intercept** | | | |  | **Random  intercept** | | | |
| **Estimation** |  | **ML** | **REML** | **REML+KR** | **REML+Satt** |  | **ML** | **REML** | **REML+KR** | **REML+Satt** |
| *Scenario** |  |  |  |  |  |  |  |  |  |  |
| **Base Case** |  | 0.40 | 0.29 | 0.38 | 0.38 |  | 0.31 | 0.32 | 0.44 | 0.46 |
| **A1** |  | 0.20 | 0.16 | 0.21 | 0.21 |  | 0.24 | 0.22 | 0.31 | 0.32 |
| **A2** |  | 1.27 | 0.81 | 1.01 | 1.03 |  | 0.44 | 0.45 | 0.75 | 0.77 |
| **B1** |  | 1.10 | 0.73 | 3.74 | 3.76 |  | 0.79 | 0.74 | 24.67 | 24.81 |
| **B2** |  | 1.11 | 0.82 | 6.27 | 7.06 |  | 0.87 | 0.81 | 59.74 | 59.19 |
| **B1-A1** |  | 0.43 | 0.34 | 1.81 | 1.84 |  | 0.48 | 0.50 | 12.67 | 13.19 |
| **B1-A2** |  | 3.47 | 2.34 | 8.24 | 8.47 |  | 1.46 | 1.42 | 48.70 | 46.61 |
| **B2-A1** |  | 0.49 | 0.46 | 3.66 | 3.67 |  | 0.59 | 0.67 | 36.32 | 36.59 |
| **B2-A2** |  | 3.06 | 2.50 | 13.59 | 13.93 |  | 1.51 | 1.49 | 124.67 | 121.51 |
| **B3** |  | 0.31 | 0.26 | 0.29 | 0.30 |  | 0.26 | 0.30 | 0.34 | 0.36 |
| **D1** |  | 0.39 | 0.28 | 0.39 | 0.40 |  | 0.52 | 0.30 | 0.47 | 0.49 |
| **D2** |  | 0.39 | 0.24 | 0.34 | 0.34 |  | 0.29 | 0.27 | 0.43 | 0.43 |

*See Table I for full data generation details relating to each scenario.

Options: ML, maximum likelihood estimation with standard confidence interval (CI) derivation; REML, restricted maximum likelihood estimation with standard CI derivation; REML+KR, REML estimation with Kenward-Roger CI derivation; REML+Satt, REML estimation with Satterthwaite CI derivation.

**Web Table C.VII.** Mean run time (in seconds) of simulations, under different scenarios for the common treatment effect data generating mechanism. Results shown separately for stratified and random intercept models, under each of the different estimation options considered.

|  |  | **Average run time, seconds** | | | | | | |
| --- | --- | --- | --- | --- | --- | --- | --- | --- |
| **Method for modelling intercept** |  | **Stratified  intercept** | |  | **Random  intercept** | | | |
| **Estimation** |  | **ML** | **REML** |  | **ML** | **REML** | **REML+KR** | **REML+Satt** |
| *Scenario** |  |  |  |  |  |  |  |  |
| **Base Case** |  | 0.03 | 0.03 |  | 0.12 | 0.10 | 0.30 | 0.22 |
| **A1** |  | 0.03 | 0.03 |  | 0.08 | 0.09 | 0.20 | 0.15 |
| **A2** |  | 0.05 | 0.05 |  | 0.16 | 0.14 | 0.58 | 0.38 |
| **B1** |  | 0.05 | 0.05 |  | 0.29 | 0.28 | 51.20 | 15.43 |
| **B2** |  | 0.04 | 0.05 |  | 0.27 | 0.27 | 92.07 | 36.86 |
| **B1-A1** |  | 0.03 | 0.03 |  | 0.17 | 0.16 | 23.15 | 7.45 |
| **B1-A2** |  | 0.07 | 0.09 |  | 0.47 | 0.49 | 97.91 | 30.43 |
| **B2-A1** |  | 0.03 | 0.04 |  | 0.19 | 0.19 | 55.24 | 20.75 |
| **B2-A2** |  | 0.08 | 0.10 |  | 0.52 | 0.53 | 183.55 | 69.07 |
| **B3** |  | 0.03 | 0.03 |  | 0.09 | 0.10 | 0.22 | 0.19 |
| **C1** |  | 0.03 | 0.03 |  | 0.10 | 0.10 | 0.32 | 0.22 |
| **C2** |  | 0.03 | 0.03 |  | 0.10 | 0.10 | 0.31 | 0.22 |

*See Table I for full data generation details relating to each scenario.

Options: ML, maximum likelihood estimation with standard confidence interval (CI) derivation; REML, restricted maximum likelihood estimation with standard CI derivation; REML+KR, REML estimation with Kenward-Roger CI derivation; REML+Satt, REML estimation with Satterthwaite CI derivation. (Note: KR and Satterthwaite correction options not available for stratified intercept model as model does not include any random effects).

**Web Table C.VIII.** Empirical SE and mean-square error (MSE) of the summary treatment effect estimate ($\hat{\theta})$, under different scenarios for the normal intercept (random treatment effect) data generating mechanism. Results shown separately for stratified and random intercept models, under each of the different estimation options considered.

| **Performance measure** |  | **Empirical SE of** $\hat{\boldsymbol{\theta}}$ | | | | |  | **MSE of** $\hat{\boldsymbol{\theta}}$ | | | | |
| --- | --- | --- | --- | --- | --- | --- | --- | --- | --- | --- | --- | --- |
|  |  |  |  |  |  |  |  |  |  |  |  |  |
| **Method for modelling intercept** |  | **Stratified intercept** | |  | **Random intercept** | |  | **Stratified intercept** | |  | **Random intercept** | |
| **Estimation** |  | **ML** | **REML** |  | **ML** | **REML** |  | **ML** | **REML** |  | **ML** | **REML** |
| *Scenario** |  |  |  |  |  |  |  |  |  |  |  |  |
| **Base Case** |  | 1.46 | 1.46 |  | 1.46 | 1.46 |  | 2.14 | 2.13 |  | 2.14 | 2.13 |
| **A1** |  | 2.04 | 2.04 |  | 2.04 | 2.04 |  | 4.15 | 4.15 |  | 4.16 | 4.15 |
| **A2** |  | 1.02 | 1.02 |  | 1.02 | 1.02 |  | 1.04 | 1.04 |  | 1.04 | 1.04 |
| **B1** |  | 1.15 | 1.14 |  | 1.15 | 1.14 |  | 1.32 | 1.31 |  | 1.32 | 1.31 |
| **B2** |  | 1.23 | 1.22 |  | 1.22 | 1.22 |  | 1.51 | 1.49 |  | 1.49 | 1.49 |
| **B1-A1** |  | 1.59 | 1.59 |  | 1.59 | 1.59 |  | 2.54 | 2.52 |  | 2.54 | 2.52 |
| **B1-A2** |  | 0.81 | 0.81 |  | 0.81 | 0.81 |  | 0.66 | 0.65 |  | 0.66 | 0.66 |
| **B2-A1** |  | 1.68 | 1.69 |  | 1.67 | 1.69 |  | 2.81 | 2.86 |  | 2.79 | 2.85 |
| **B2-A2** |  | 0.86 | 0.85 |  | 0.85 | 0.84 |  | 0.74 | 0.72 |  | 0.72 | 0.71 |
| **B3** |  | 1.78 | 1.78 |  | 1.78 | 1.78 |  | 3.16 | 3.17 |  | 3.16 | 3.17 |
| **C1** |  | 1.46 | 1.46 |  | 1.46 | 1.46 |  | 2.14 | 2.14 |  | 2.14 | 2.14 |
| **C2** |  | 1.46 | 1.46 |  | 1.46 | 1.46 |  | 2.13 | 2.14 |  | 2.13 | 2.14 |
| **D1** |  | 1.32 | 1.32 |  | 1.32 | 1.32 |  | 1.74 | 1.74 |  | 1.74 | 1.74 |
| **D2** |  | 1.71 | 1.71 |  | 1.71 | 1.71 |  | 2.91 | 2.92 |  | 2.91 | 2.92 |

*See Table I for full data generation details relating to each scenario.

Options: ML, maximum likelihood estimation; REML, restricted maximum likelihood estimation.

**Web Table C.IX.** Empirical SE and mean-square error of the summary treatment effect estimate ($\hat{\theta})$, under different scenarios for the beta distribution for the intercept (random treatment effect) data generating mechanism. Results shown separately for stratified and random intercept models, under each of the different estimation options considered.

| **Performance measure** |  | **Empirical SE of** $\hat{\boldsymbol{\theta}}$ | | | | |  | **MSE of** $\hat{\boldsymbol{\theta}}$ | | | | |
| --- | --- | --- | --- | --- | --- | --- | --- | --- | --- | --- | --- | --- |
|  |  |  |  |  |  |  |  |  |  |  |  |  |
| **Method for modelling intercept** |  | **Stratified intercept** | |  | **Random intercept** | |  | **Stratified intercept** | |  | **Random intercept** | |
| **Estimation** |  | **ML** | **REML** |  | **ML** | **REML** |  | **ML** | **REML** |  | **ML** | **REML** |
| *Scenario** |  |  |  |  |  |  |  |  |  |  |  |  |
| **Base Case** |  | 1.83 | 1.84 |  | 1.83 | 1.84 |  | 3.36 | 3.40 |  | 3.36 | 3.40 |
| **A1** |  | 2.72 | 2.71 |  | 2.72 | 2.71 |  | 7.39 | 7.35 |  | 7.39 | 7.34 |
| **A2** |  | 1.30 | 1.30 |  | 1.30 | 1.30 |  | 1.68 | 1.68 |  | 1.68 | 1.68 |
| **B1** |  | 1.32 | 1.33 |  | 1.32 | 1.33 |  | 1.75 | 1.78 |  | 1.74 | 1.77 |
| **B2** |  | 1.36 | 1.35 |  | 1.32 | 1.32 |  | 1.85 | 1.83 |  | 1.74 | 1.74 |
| **B1-A1** |  | 1.99 | 1.98 |  | 1.99 | 1.98 |  | 3.97 | 3.93 |  | 3.95 | 3.92 |
| **B1-A2** |  | 0.97 | 0.97 |  | 0.97 | 0.97 |  | 0.94 | 0.94 |  | 0.94 | 0.94 |
| **B2-A1** |  | 1.80 | 1.80 |  | 1.76 | 1.73 |  | 3.24 | 3.26 |  | 3.09 | 3.00 |
| **B2-A2** |  | 0.98 | 0.97 |  | 0.95 | 0.94 |  | 0.97 | 0.93 |  | 0.90 | 0.89 |
| **B3** |  | 2.40 | 2.40 |  | 2.37 | 2.37 |  | 5.77 | 5.76 |  | 5.65 | 5.64 |
| **D1** |  | 1.72 | 1.73 |  | 1.72 | 1.73 |  | 2.97 | 2.99 |  | 2.97 | 2.99 |
| **D2** |  | 2.04 | 2.03 |  | 2.04 | 2.03 |  | 4.16 | 4.11 |  | 4.16 | 4.11 |

*See Table I for full data generation details relating to each scenario.

Options: ML, maximum likelihood estimation; REML, restricted maximum likelihood estimation.

**Web Table C.X.** Empirical SE and mean-square error of the summary treatment effect estimate ($\hat{\theta})$, under different scenarios for the common treatment effect data generating mechanism. Results shown separately for stratified and random intercept models, under each of the different estimation options considered.

| **Performance measure** |  | **Empirical SE of** $\hat{\boldsymbol{\theta}}$ | | | | |  | **MSE of** $\hat{\boldsymbol{\theta}}$ | | | | |
| --- | --- | --- | --- | --- | --- | --- | --- | --- | --- | --- | --- | --- |
| **Method for modelling intercept** |  | **Stratified intercept** | |  | **Random intercept** | |  | **Stratified intercept** | |  | **Random intercept** | |
| **Estimation** |  | **ML** | **REML** |  | **ML** | **REML** |  | **ML** | **REML** |  | **ML** | **REML** |
| *Scenario** |  |  |  |  |  |  |  |  |  |  |  |  |
| **Base Case** |  | 1.11 | 1.11 |  | 1.11 | 1.11 |  | 1.25 | 1.25 |  | 1.24 | 1.24 |
| **A1** |  | 1.68 | 1.68 |  | 1.68 | 1.68 |  | 2.81 | 2.81 |  | 2.81 | 2.81 |
| **A2** |  | 0.83 | 0.83 |  | 0.83 | 0.83 |  | 0.68 | 0.68 |  | 0.68 | 0.68 |
| **B1** |  | 0.71 | 0.71 |  | 0.71 | 0.71 |  | 0.50 | 0.50 |  | 0.50 | 0.50 |
| **B2** |  | 0.49 | 0.49 |  | 0.49 | 0.49 |  | 0.24 | 0.24 |  | 0.24 | 0.24 |
| **B1-A1** |  | 0.95 | 0.95 |  | 0.95 | 0.95 |  | 0.90 | 0.90 |  | 0.90 | 0.90 |
| **B1-A2** |  | 0.53 | 0.53 |  | 0.53 | 0.53 |  | 0.28 | 0.28 |  | 0.28 | 0.28 |
| **B2-A1** |  | 0.69 | 0.69 |  | 0.69 | 0.69 |  | 0.47 | 0.47 |  | 0.47 | 0.47 |
| **B2-A2** |  | 0.36 | 0.36 |  | 0.36 | 0.36 |  | 0.13 | 0.13 |  | 0.13 | 0.13 |
| **B3** |  | 1.52 | 1.52 |  | 1.52 | 1.52 |  | 2.32 | 2.32 |  | 2.32 | 2.32 |
| **C1** |  | 1.11 | 1.11 |  | 1.11 | 1.11 |  | 1.25 | 1.25 |  | 1.24 | 1.24 |
| **C2** |  | 1.11 | 1.11 |  | 1.11 | 1.11 |  | 1.25 | 1.25 |  | 1.24 | 1.24 |

*See Table I for full data generation details relating to each scenario.

Options: ML, maximum likelihood estimation; REML, restricted maximum likelihood estimation.
